# Articulating current service development practices: a qualitative analysis of eleven mental health projects

**DOI:** 10.1186/1472-6963-14-20

**Published:** 2014-01-17

**Authors:** Gyuchan Thomas Jun, Cecily Morrison, P John Clarkson

**Affiliations:** 1 Loughborough Design School, Loughborough University, Loughborough LE11 3TU, UK; 2 Engineering Department, University of Cambridge, Trumpington Street, Cambridge CB2 1PZ, UK

## Abstract

**Background:**

The utilisation of good design practices in the development of complex health services is essential to improving quality. Healthcare organisations, however, are often seriously out of step with modern design thinking and practice. As a starting point to encourage the uptake of good design practices, it is important to understand the context of their intended use. This study aims to do that by articulating current health service development practices.

**Methods:**

Eleven service development projects carried out in a large mental health service were investigated through in-depth interviews with six operation managers. The critical decision method in conjunction with diagrammatic elicitation was used to capture descriptions of these projects. Stage-gate design models were then formed to visually articulate, classify and characterise different service development practices.

**Results:**

Projects were grouped into three categories according to design process patterns: new service introduction and service integration; service improvement; service closure. Three common design stages: problem exploration, idea generation and solution evaluation - were then compared across the design process patterns. Consistent across projects were a top-down, policy-driven approach to exploration, underexploited idea generation and implementation-based evaluation.

**Conclusions:**

This study provides insight into where and how good design practices can contribute to the improvement of current service development practices. Specifically, the following suggestions for future service development practices are made: genuine user needs analysis for exploration; divergent thinking and innovative culture for idea generation; and fail-safe evaluation prior to implementation. Better training for managers through partnership working with design experts and researchers could be beneficial.

## Background

The utilisation of good design practices to develop complex health services is essential to their quality [1-4]. Good design practices can be defined as methods that lead to design artifacts, either products or services, having a ‘fitness for purpose’ for their task while maintaining commercial viability [5]. The establishment of good design practices has come from 50 years of study and reflection across a range of sub-disciplines, including industrial design, product design and engineering design [6].

Service development, often referred to as service design, is an on-going activity that helps healthcare organisations meet a changing kaleidoscope of new challenges and opportunities. For example, changes in services are required to meet increased demand under budget constraints or integrate new technologies. Many healthcare organisations have sought to emulate operation principles that lead to highly reliable and safe practices [7].

Healthcare organisations, however, are often considered seriously out of step with modern design thinking and practice, particularly in relation to patient safety [8,9]. This is not due to a lack of health care-specific tools. A large number of design tools and methods have been introduced to healthcare organisations [7,10-13]. Yet, many remain at a conceptual level and have not been utilised to the same degree as in other sectors such as automotive and aerospace [8,14].

The proactive utilisation of good design practices in other domains indicates that adoption is increased if the context of intended use is understood [6]. To this aim, the study presented in this paper articulates current service development practices. Description of existing practices can provide insight into how healthcare leaders and managers might better utilise good design practices in health service development projects.

## Methods

### Study context

The study took place in Cambridgeshire and Peterborough NHS foundation trust in the United Kingdom. The trust is a designated Cambridge University Teaching Trust and provides mental health services to 755,000 residents. The research team gained access to an older people’s division in the trust as a part of a larger applied health research project (CLAHRC: Collaboration for Leadership in Applied Health Research and Care) funded by National Institute of Health Research (NIHR), UK.

### Participants

All six operation managers in the older people’s mental health division participated in the study. Four were regional operation managers, one a service development manager, and one a senior manager. All had previous roles in either health or social care provision, with four being former nurses, one a former occupational therapist, and one a former social worker. They had between 15 and 30 year experience in health or social care services each, including 5 to 20 years in management. One of their main management roles had been to lead or coordinate service development projects.

### Data collection

**Figure 1:**
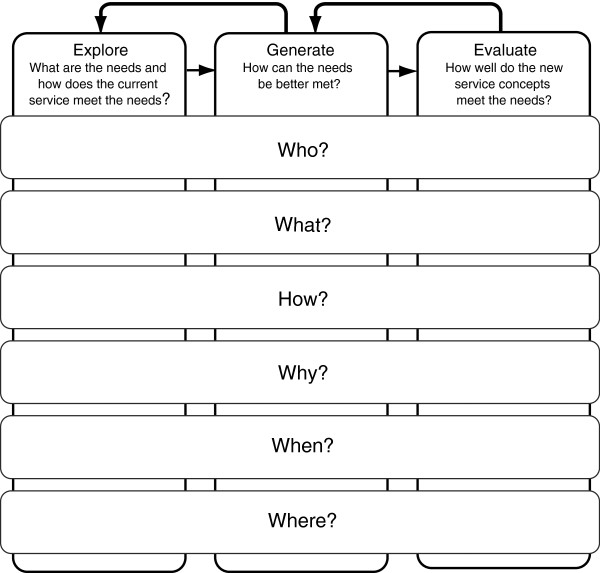
Interview guides – what happened in your previous project at each of the three design stages?

The study uses a retrospective semi-structured interview strategy to investigate various aspects of service development practices. Principles from the critical decision method are adopted [15]. This approach has been used successfully to investigate the cognitive bases of judgement and decision-making of an individual in naturalistic settings in environments from system development to intensive care units [16,17]. This study draws specifically on the following elements: a case-based approach, a focus on non-routine cases, semi-structured probing, and cognitive probes [15]. Diagram-based cues (Figure 1) are used to facilitate decision point probing.

Diagrammatic elicitation helps to capture a more detailed recollection of activities and decisions than questions alone [18,19]. A diagram, adapted from Cross [20], representing a simple three stage design process is used (Figure 1). The three stages are: explore (what are the needs and how are these met through current services?), generate (how can the needs be better met?) and evaluate (how well do the new service concepts meet the needs?). Each stage is further probed through more specific cues: who; what; how; why; when; where. These are represented by the rows in Figure 1.

Participants were contacted by email a week before the interviews and asked to choose two service development projects to discuss. It was specified that they should have been carried out within the past five years and that one should be considered successful and the other, unsuccessful. Our intention was to obtain more specific information by probing concrete and non-routine cases as argued in the critical decision method [15].

Relevant background information to the project was captured at the beginning of the interviews. This included personal information such as current role, years of experience, and training and qualifications, as well as project information such drivers, goals and duration. The diagram (Figure 1) was then placed on the table as a visual prompt and the series of cue questions for each design stage were asked in relation to the projects they chose. The diagram and questions provided prompts, but the participants were allowed to discuss their projects as they wished. The audio-recorded interviews took between 1 and 1.5 hours and were transcribed.

### Analysis

The transcribed interviews were initially coded using a framework derived from the elicitation diagram (Figure 1). Relevant data were captured for each design stage in tabular form. Columns included: who was involved, what actions or decisions were taken, how were decisions made, as well as why, when and where. Projects were then characterised and grouped by *goals, drivers, durations* and *design activities*. During this initial analysis, the authors identified that the majority of the projects were approval-driven. For example, the progress of many design activities was controlled by approvals from committees, senior management or commissioners.

Stage-gate design process models were chosen to further visualise the project narratives. Stage-gate models break a design project into sets of stages and gates. Each stage consists of a set of activities and a gate is an entrance to a stage. Gates serve as quality control mechanisms and check points [21]. Stage gate models have been adopted by many technology companies to better understand and manage their project development processes [21]. In this study, we depicted the narratives according to such models in order to support consistent comparison between projects and recognise distinctive patterns. The various design activities of each project were categorised into the design stages (explore, generate and evaluate) as defined in Figure 1. Gates were then identified between stages.

This study did not require review by a NHS research ethics committee as it was classified as service evaluation. It was reviewed and approved by local NHS research governance (Cambridgeshire and Peterborough NHS foundation trust).

## Results

Eleven projects were identified. The features of each project in terms of *goals, drivers, durations* and *design activities* are described in Table 1.

**Table 1:** Summary descriptions of the eleven projects

**Goal**	**Project**	**Driver**	**Duration**	**Summary**
New service introduction	1. Introduction of memory services to primary care	Government policies	9 months	A project team was set up to pilot a new service model (memory clinic service in primary care) proposed from government policies. A business case was developed to get approval from a commissioning body and two days a week piloting was carried out.
		GPs’ complaints		
	2. Roll-out of a primary care psychology service	Cost saving	18 months	A multidisciplinary working group was set up to conduct a full-scale implementation of a new service after piloting. After the review of the piloting outcomes, detailed implementation plans were developed to get an approval from executive committee and a commissioning body.
		Demand growth		
	3. Introduction of an intermediate mental health care team	GPs’ complaints	2 years (piloting) 3 years (roll-out)	A working group was set up to introduce a new intermediate care team. A proposal for five days a week piloting was developed, approved by a commissioning body and conducted. Then, a roll-out planning was developed, approved and conducted.
		Government policies		
	4. Development of a new mental health unit building	Population growth	8 years	A core project team was set up to conduct a new building development project. Benchmarking and evidence search was to capture requirements for the new building. Architects were involved to develop drawings and mock-ups based on which various evaluations (accessibility, noise, layout, supplies, etc.) were carried out.
Service integration	5. Integration between social and health care teams (region A)	Government policies	1 year (piloting)	A project lead was appointed to implement a team integration piloting project. Team social events were organised to address staff’s concern. A partnership agreement was developed to conduct six month piloting.
	6. Integration between social and mental health care teams (region B)	Government policies	2 years (piloting)	A project lead was appointed to lead a full scale implementation of a team integration project. Detailed service models and implementation plans were developed and approved by a steering committee.
Service improvement	7. Interface improvement between health care and social care	Government policies	6 months	A project working group was appointed to improve service interface between social and health care. Staff workshops were arranged to develop new service concepts and agreement was made. Implementation was not approved by a commissioning body.
	8. Lean service transformation	Senior management	12 months	An external consultancy was appointed to conduct a lean service transformation project. Staff workshops were organised along with lean methodology training. Seven rapid improvement activities were proposed, approved by a project board and implemented.
		Cost saving		
		Better efficiency		
	9. Review and redesign of physiotherapy services	Staff retirement	12 months	A project lead was appointed to develop a service improvement project. Current services were mapped and staff members were interviewed. An improvement proposal was developed and approved by senior management and implemented.
		Cost saving		
	10. Interface improvement between primary and secondary mental health care	GPs’ complaints	2 years	A working group was appointed to improve service interface between primary and secondary care. Staff workshops were organised to understand current system and develop new concept solutions. The implementation was approved by a committee.
Service closure	11. Close-down of care home services	Financial loss	6 months	A project team including a communication lead was appointed to implement service closure. Individual patient’s needs were assessed to determine care plans after closure. Public and staff consultations were arranged. The project was completed by the post closure check by a local council.

### Characterisation of the projects

Four different *project goals* were identified: new service introduction; service integration; service improvement (redesign of existing services); and service closure. Four new service introduction projects (1, 2, 3 and 4 in Table 1) aimed to implement a new service model or develop a new building; two service integration projects (5 and 6 in Table 1) endeavoured to integrate two care delivery teams into one; four service improvement projects (7, 8, 9 and 10 in Table 1) aspired to improve existing services by making them leaner, more efficient or cost-effective. One service closure project (11 in Table 1) closed down a financially-unsustainable service.

**Figure 2:**
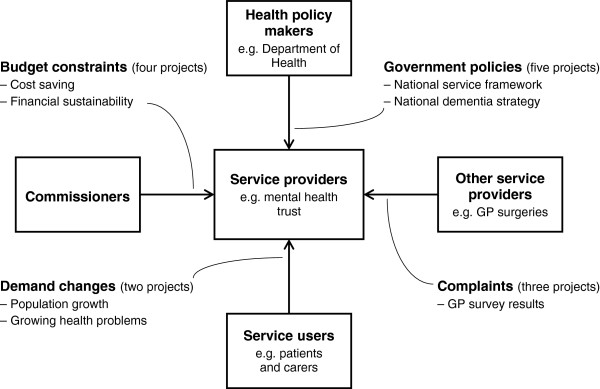
Drivers of service development projects.

Four key *drivers* for project initiation were identified: government policies (1, 3, 5, 6 and 7 in Table 1); service demand (2 and 4 in Table 1); complaints from other service providers (1, 3 and 10 in Table 1); and budget constraints (2, 8 and 11 in Table 1). These are illustrated in Figure 2. New strategies and service models in government policies such as National Service Frameworks [22,23] and the National Dementia Strategy [24] played a substantial role in initiating five of the service development projects identified in this study. Service demand increase from regional population growth or increasing prevalence of certain health problems instigated two projects. Complaints from other service providers, such as the annual GP survey, droved three projects. Budget constraints initiated two service improvement projects and continuous deficit of one service triggered its closure. Although we have described important catalysts for projects, they are not distinct categories and most had more than one.

In terms of *duration*, the majority of projects spanned six months to two years. Two lasted longer than five years (3 & 4 in Table 1). The *scope* of the projects ranged from early exploration of ideas to full-scale implementation.

Based on the characterisation of the projects, three stage-gate models were generated. These represent distinctive service development practices: new service introduction and service integration projects; service improvement projects; and service closure project. The new service introduction projects and the service integration projects, although having different project goals, were similar in project structure and thus grouped together. An implementation stage, which was discovered to account for a substantial part of the service development projects in this study, was added to the Cross’ three design stage model [20].

### New service introduction and service integration projects

**Figure 3:**
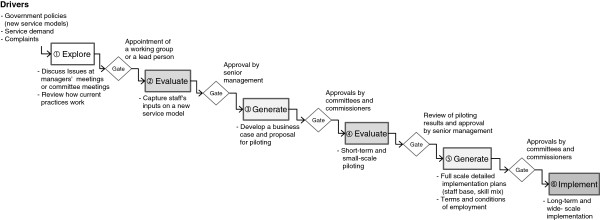
New service development and service integration project.

Figure 3 shows the stage gate model of the new service introduction and the service integration projects. The building development project (4 in Table 1), which has its unique architect-driven process, was excluded from this model. Various design activities were categorised according to four general design stages (explore, generate, evaluate and implement).

Service introduction or integration projects were driven frequently by new government policy documents. New mandates were initially discussed in regular management meetings and gaps between existing service delivery practices and the newly propose service model were *explored* briefly (① in Figure 3). Following on from this, a working group or project lead would be appointed depending on the nature of the project. A team or lead individual then started the ‘*evaluate*’ stage (② in Figure 3) by informally gathering staff’s views and concerns of the new policy. Then senior management approved the need for business case development. Business cases for pilot projects were *generated* and approved by committees and commissioners (③ in Figure 3).

Piloting was carried out as a way of *evaluating* new service models. The duration of the pilot projects ranged from 6 to 18 months. Duration normally depended on the nature of project and funding available (④ in Figure 3). Pilot projects were by their nature carried out on a limited scale, usually with designated team(s). After the piloting, the results were reviewed and the approval to full scale implementation planning was made by senior management. The *generation* of a full scale implementation plan (⑤ in Figure 3) took another series of work group meetings. In these meetings, decisions relating to the details of implementation were taken, such as, staff base, skill mix, and terms and conditions of employment. Approval by committees and commissioners had to be obtained before long-term and wide-scale *implementation* (⑥ in Figure 3) could take place.

### Service improvement projects

**Figure 4:**
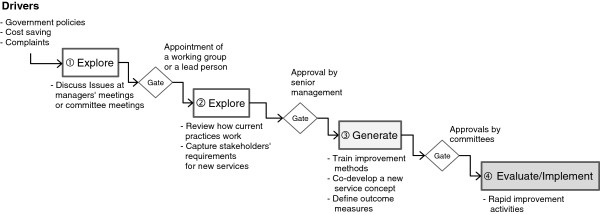
Service improvement project.

Figure 4 shows the stage gate model of the service improvement projects. The first two ‘*explore*’ stages are similar to Figure 3. Unlike new service introduction and integration projects, in which the scope for new idea generation is rarely possible, there is an explicit ‘new idea generation’ stage (③ in Figure 4).

During the ‘*generate*’ stage, project teams ran stakeholder workshops in which invited healthcare professionals and managers co-created new service concepts. In workshops, they usually introduced and applied various design methods such as process mapping and lean principles to support analysis and idea generation. Rapid improvement activities rather than piloting were mentioned as mechanisms for *evaluation*. These focused on the *implementation* (④ in Figure 4) of small changes. Given the relatively small project scale and unlikely need for a large amount of funding, commissioners were rarely involved in the final approvals of the service improvement projects.

### Service closure projects

**Figure 5:**
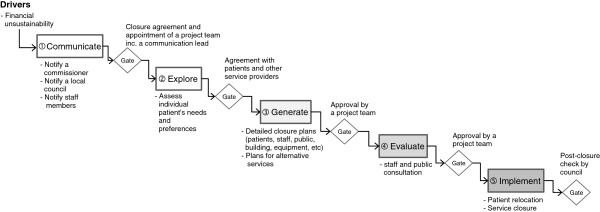
Service closure project.

Figure 5 shows the stage gate model of the service closure project. The service closure, although there is just one case in this study, shows very distinctive process patterns. The service closure was driven by financial loss over several years with no sign of improvement. Although one service closure project was identified and described in this study, two types of service closure were discussed during the interview: the major service closure which is likely to affect the interest of general public; the minor service closure that would not affect the interest of general public. The process description in Figure 5 is a major service closure case which requires the local council’s involvement and public consultation.

The service closure project was characterized as being communication-critical. Not only formal communication (notifications and consultations), but also informal communication with staff, patients, public, local council committee and other service providers were described as essential. Communicating the right issues, at the right time, with the right people, was considered the key to a smooth service closure. The project started with a *communication* stage where a service provider gave advance notices to relevant stakeholders (① in Figure 5). Then, a project team was formed from both the commissioning body and relevant service providing bodies. Unlike the other projects, a communication lead was appointed at a very early stage. This person played an important role, communicating with various stakeholders: patient, public, staff, local council and media. Given the sensitive and serious nature of this type of project, how, what, when and with whom to communicate were considered very important.

The main task at the first *explore* stage was to assess each patient to identify their needs and preferences for alternative service provision (② in Figure 5). Based on this assessment, detailed closure plans were *generated* (③ in Figure 5). The overall closure plans were *evaluated* in public and staff consultations events (④ in Figure 5) and eventually *implemented* (② in Figure 5). The closure project ended with the post-closure check by a local council committee member several months later.

## Discussion

Based on current service development practices this study articulated, we would like to discuss how good design practices could be utilised at each design stage to increase the likelihood of successful implementation.

### Explore

‘Exploration’ in design processes means to explore the ill-defined problem space before generating a concept solution [20]. It could be asking questions about genuine needs and problems or investigating how well the current solutions meet those needs or address those problems. In most of the health service development processes of this study, this stage was present, but not used to full benefit.

In new service development and integration projects, not only problems, but also concept solutions, i.e. new service models, are most frequently provided in policy documents. Indeed, multiple interviewees alluded to the necessity of aligning project goals to government policies to win approval and financial support. For example, a service introduction project (3 in Table 1) was initiated by a manager to address chronic service interface problems. It failed to gain approval until the proposal was revised to reference a new government policy two years later. This example illustrates what seemed to be a pervasive top-down approach to service design and as such, innovation.

Such an approach does not allow sufficient opportunities for stakeholders, i.e. frontline healthcare professionals, to explore problems together at the early stage of design. This decreases the likelihood of successful adoption and ownership of new ways of working [25,26]. The UK’s National Program for IT (NPfIT), which aimed to build a central electronic record that all healthcare providers across the country would use, is a visible case in point with high levels of rejection at the local level. The generalised approach had limited the capacity of healthcare organisations to adapt quickly to the dynamic and site-specific health service delivery challenges they faced [27].

These observations suggest that the ‘explore’ stage should be more explicitly conducted. Personas and stakeholder maps are illustrative examples that can support this stage. Personas are fictional profiles that describe the goals and observed behaviour patterns of a range of potential users. As a tool, they summarise user diversity, helping those discussing ‘stand in the shoes’ of multiples users [28]. Stakeholder maps are visual representation of the various groups involved with a particular service and the interplay between them [29]. They are useful in highlighting and discussing potential conflicts or interface issues. Both of these methods provide concrete representations to support a shared perspective when discussing alternatives. This is essential to enable the exploration among groups with very different experiences, such as a patient and health service manager [30].

### Generate

‘Generation’ in design processes means to generate the widest possible range of options [20]. In this sense, an idea generation stage does not exist in the new service development and service integration projects of this study. The two ‘generate’ stages in Figure 3 are not about generating a wide range of options, but rather detailed plans for implementation. There was a more explicit stage of idea generation in service improvement projects, in which problem analysis and idea generation were carried out using methods such as, lean principles and process maps.

There are a broader range of divergent thinking methods that could be used at this stage. These include fresh eyes, breaking the rules, random words and mental benchmarks [31]. Fresh eyes, for example, asks participants to take the alternative viewpoint, such as a 6 year old child, and asks how they would see the problem. These methods help alter our underlying mental models by challenging the usual stream of thought and making creative connection with fundamentally different examples [31].

However, such idea generating methods only work in organisations that develop and nurture a culture of creativity and innovation. This is at odds with the current top down policy-driven approach to service development as it undervalues stakeholder involvement and decreases intrinsic motivation. There is considerable evidence to show that intrinsic motivation is more powerful in driving up levels of creativity than extrinsic motivators, such as competitions, expected evaluation, or rewards [32]. Incorporating a culture of idea generation, and possibly innovation, in a target-driven, performance managed healthcare system is a major challenge [33].

Healthcare organisations, therefore, should think carefully not only how to exploit various divergent thinking methods, but also how to develop and nurture innovative culture in the long term. Healthcare managers, while attending to policy changes, can also consider what they might do to support an innovative culture. Previous research has shown that organisational support (e.g. time, resources, training, skills) and management support (e.g. attentiveness, coaching, giving useful feedback, being open to criticism) are also critical to the enhancement of innovative culture in health care [34,35].

### Evaluate

‘Evaluation’ in design processes means to assess a wide range of ideas against the goals and constrains of project in order to decide upon the most appropriate final concept [20]. Four types of evaluation were observed in this study: staff input, piloting, rapid improvement, and public consultation. New service development and service integration projects usually started with capturing staff’s concerns on new service models proposed in policy documents. Then, small-scale and short-term piloting was carried out. Service improvement projects had rapid improvement in which new ideas were quickly tested, adjusted and implemented into practice. Service closure projects had staff and public consultation as a formal mechanism for gaining input from affected stakeholders.

The focus of the evaluation methods seen in this study, mainly use some form of controlled implementation. This is in line with a common approach to service development in healthcare organisations, Plan-Do-Study-Act [36]. While this is appropriate to the complex nature of some types of healthcare services, it is not the only approach to evaluation and can be problematic in some circumstances. For example, studies have shown this approach to evaluating medication management in acute care led to work-arounds of emergent issues which compromised patient safety [37].

Alternative approaches provide opportunities for staff to experiment with new ideas and solutions before implementation to identify and address issues that may emerge [38,39]. Such approaches, commonly used in other safety critical industries, can also be systematic [20,21,40]. Medical device design processes [5,41] are a case in point. Varying physical, virtual and conceptual evaluations are carried out before implementation. Examples include types of system analysis, including: fault tree analysis, worst case analysis, what-if analysis and failure mode and effect analysis [42]. Computer-based scenario simulation approaches can also be used to evaluate potentially high-risk concepts prior to implementation [43].

Service design, an emerging design discipline, also has adopted various quick-and-dirty methods to collaboratively make sure that their conceptual solutions are feasible and potential risks are acceptable. Desktop walkthrough is a way of acting out ideas for service interaction with a small-scale 3-D model. Similarly, service prototyping is done by observing users interact with the materials of a service. Maps and models are also used to conceptually check flows and interactions [29,44].

These methods check the validity and safety of concepts. They also have the added benefit of allowing staff to experiment with new ideas and solutions with no risk to patients or the organisation [38,39]. This can support both more innovative solutions as well as decrease the risk of staff resistance during implementation. These approaches complement the Plan-Do-Study-Act cycle of rapid improvement [36], in situations in which an implementation-based evaluation can cause clinical safety issues.

### Limitations

Some limitations of the present research should be noted. The participants and projects were derived from one division of one regional mental health system which may limit generalizability. In addition, semi-structured interviews, although often applied to study challenges around design processes [45], could bring more certainty if combined with other methods, in this case, observations [45-47]. However, most of the authors’ anecdotal observatory experiences in other healthcare settings and projects over the last decade coincide with the findings of this study.

## Conclusion

This study set out to fill a gap in our understanding of health service development practices by examining the design processes and methods utilised in eleven mental health service development projects. This study has shown a wide gap between healthcare service development practices and modern design thinking and practices. As part of this assessment, we have highlighted appropriate methods that could be employed to close this gap.

More specifically, the study has shown that currently, there is a top-down approach to service design led by government policies. This has led to a focus on location-specific implantation rather than the generation of new service design ideas. Unfortunately, top-down approaches undervalue the exploration of genuine needs and problems through stakeholder involvement. In turn, this limits opportunities to apply more divergent thinking methods and develop an innovative, creative workforce within healthcare organisations.

The study has also demonstrated that the evaluation methods used are primarily implementation-based. Alternative methods discussed in this paper include various conceptual and virtual methods for checking validity and safety prior to implementation. These have the added benefit of allowing staff to experiment with new ideas and solutions with no risk to patients or the organisation.

Partnership working between health service managers and design researchers can contribute to finding the most appropriate design practices for the current context of health service development. These must address the unique organisational culture and limited availability of staff’s time and skill. Further research on how design practices are to be better supported in healthcare organisations is also needed. Partnerships would also enable healthcare managers to be better trained in good design practices through an appropriate, ‘learning by doing’ method. This bilateral approach will help to bring the healthcare sector in line with, and benefit from, modern design thinking and practices.

## Competing interests

The authors declare that they have no competing interests.

## Authors’ contributions

GJ conceived and designed the study, carried out data collection and analysis, and drafted and revised the manuscript. CM participated in the conception and the design of the study and helped to draft, restructure and revise the manuscript. PJC contributed to the conception of the study and helped to restructure the manuscript as well. All authors read and approved the final manuscript.

## Pre-publication history

The pre-publication history for this paper can be accessed here:

http://www.biomedcentral.com/1472-6963/14/20/prepub
